# Chinese Writing of Deaf or Hard-of-Hearing Students and Normal-Hearing Peers from Complex Network Approach

**DOI:** 10.3389/fpsyg.2016.01777

**Published:** 2016-11-22

**Authors:** Huiyuan Jin, Haitao Liu

**Affiliations:** ^1^Department of Linguistics, School of International Studies, Zhejiang UniversityHangzhou, China; ^2^Department of Foreign Languages, Ningbo Institute of Technology, Zhejiang UniversityNingbo, China

**Keywords:** deaf or hard-of-hearing students, normal-hearing peers, language system, Chinese writing, complex network theory

## Abstract

Deaf or hard-of-hearing individuals usually face a greater challenge to learn to write than their normal-hearing counterparts. Due to the limitations of traditional research methods focusing on microscopic linguistic features, a holistic characterization of the writing linguistic features of these language users is lacking. This study attempts to fill this gap by adopting the methodology of linguistic complex networks. Two syntactic dependency networks are built in order to compare the macroscopic linguistic features of deaf or hard-of-hearing students and those of their normal-hearing peers. One is transformed from a treebank of writing produced by Chinese deaf or hard-of-hearing students, and the other from a treebank of writing produced by their Chinese normal-hearing counterparts. Two major findings are obtained through comparison of the statistical features of the two networks. On the one hand, both linguistic networks display small-world and scale-free network structures, but the network of the normal-hearing students' exhibits a more power-law-like degree distribution. Relevant network measures show significant differences between the two linguistic networks. On the other hand, deaf or hard-of-hearing students tend to have a lower language proficiency level in both syntactic and lexical aspects. The rigid use of function words and a lower vocabulary richness of the deaf or hard-of-hearing students may partially account for the observed differences.

## Introduction

Humans acquire language in a diverse set of circumstances. Normal-hearing (NH) individuals first learn a spoken language, which is followed by reading and writing. In contrast to the auditory-vocal modality of spoken language, the visual-gestural modality of sign languages is often used among deaf or hard-of-hearing (DHH) individuals (Stokoe, 2003; Leonard et al., 2013). Sign language is very different from written and spoken language in that it is composed of signs corresponding to locations and movements along with facial expressions and body gestures (Emmorey, 2001; Lu et al., 2015). Chinese sign language is different from Chinese spoken language in various linguistic aspects, such as phonology, morphology, lexicon, syntax, and pragmatics, etc. (Wu, 2005, 2006; Gong, 2009). For instance, modern Chinese is generally considered as an example of analytic language, whereas Chinese sign language uses visually-based complex inflectional and derivational systems to encode aspect, spatial relationships and to form classes, etc. The smallest semantic unit is Chinese characters and they are used in linear sequences to express meaning (Yu and Zhang, 2004; Liu, 2005; Lederberg et al., 2013). Word order is usually fixed and the SVO structure is the predominant sentence pattern in modern spoken Chinese. However, multiple syntactic features may be simultaneously represented by hands, facial expressions, and different types of body movements in Chinese sign language. Therefore, word order of Chinese sign language is rather flexible compared with that of Chinese spoken language (Liu, 2005; Wu, 2006; Gong, 2009). For DHH individuals whose primary means of communication is sign language, learning to read and write is essentially the same as learning a second language (Emmorey, 2001; Stokoe, 2003). Therefore, the DHH children usually face a substantially larger challenge learning to read and write than their NH counterparts.

Reading and writing abilities are two primary literacy abilities for DHH individuals (Evans, 2004; Lederberg et al., 2013). But this study will specifically pertain to the writing of Chinese deaf group. Various research has discussed that for the majority of DHH students, learning to write is a tortuously slow and frustrating process (Luckner et al., 2005). Their written language has been described as “concrete, repetitive, and structurally simplistic” (Marschark, 1997). Syntactic aspects of DHH students have also been studied, including passive constructions, subject-verb-object word order, and relative clauses (Power and Quigley, 1973; Quigley et al., 1974a,b; Bochner, 1978; Quigley and King, 1980; Berent, 1988). Antia et al. (2005) observe that DHH children are behaving poorer in vocabulary and syntax compared to their hearing peers. Wu (2005) notes that due to the grammatical differences across sign language and Chinese written language, adjectives, adverbs, and conjunctions are usually absent in the writing of DHH students; the frequency of some syntactic structures is significantly different in comparison to that of hearing people, and their writing is less cohesive and less elaborate. Quigley and Paul (1984) state that DHH students are more likely to produce shorter sentences and avoid complex syntactic structures. The combined results suggest that the language systems of the DHH and hearing students are roughly alike, but a comparatively lower language proficiency level of writing is often found in the former.

However, some other researchers may hold rather radical views on the written language of DHH students. These researchers have asserted that DHH students have poor or no linguistic competence, or that they have difficulties in understanding and producing sentences. The compositions of DHH students have been described as having “a simpler style, involving relatively rigid, unrelated language units which follow each other with little overlapping or structure or meaning” (Heider and Heider, 1941). DHH students show differences not merely in skills in the syntactic structures, but also in the whole thought structure (Heider and Heider, 1941). These combined results point to the conclusion that the writing of DHH students is frequently very deviant from the language produced by hearing people (Ivimey, 1976). In other words, the language systems of DHH students and NH students may be totally different.

In sum, two different views co-exist concerning the writing system and the writing ability of DHH students. On the one hand, it is assumed by some that the language of DHH students, although somewhat restricted and defective, is similar to that of NH students. On the other hand, there are beliefs that DHH students use a totally different system of language rules to understand and produce sentences. These views are contradictory and irreconcilable. There is a need to solve this issue with appropriate methodology.

Previous investigations on language features of the writing of DHH students, be they quantitative or qualitative, have all been descriptive; they have focused only on the detailed linguistic features and therefore lack insights into the macroscopic properties. The macroscopic properties of language can be hardly captured by traditional descriptive approaches. That being so, complex network approach is introduced and used in this study. This new research approach provides opportunities to investigate language systems from a macroscopic perspective, which is a necessary complement to the wealth of findings concerning the micro structure of human language (Cong and Liu, 2014). The quantitative network analysis of language sub-systems, such as the syntactic sub-system of DHH students' Chinese writing, provides a characterization of the complex organization and thus a macro structure. With the complex network approach, a comparative study of the syntactic sub-systems of Chinese DHH students and their NH peers is conducted. The syntactic dependency networks are adopted as models for the syntactic sub-system of the two groups. Based upon the two networks, this study attempts to address the following two research questions:

1. What are the overall differences of the syntactic sub-systems of the Chinese DHH students and their NH peers from a complex network perspective?
2. What are the differences of the DHHs and the NHs in the linguistic competence of writing as shown by comparison of specific measures of the two network models?

Question 1 is intended to reveal the quantitative features of the two syntactic dependency networks. Question 2 is trying to discover the specific differences of the two networks and relevant underlying linguistic implications in terms of writing. These two questions will help to understand the macroscopic features of the syntactic sub-system of DHH students' Chinese writing; and further, the overall discrepancies of linguistic competence of writing between the two groups. Details of the language materials and research methods will be introduced in Section Materials and Methods. Section Results will provide the relevant results. Relative discussions following the observations and comparisons of network measures will be presented in Section Discussions.

## Materials and methods

### The construction of syntactic dependency networks

A network is a set of items, which we call vertices (or nodes), with connections between them, called edges (Newman, 2003, 2010). In a language network, vertices could be linguistic units of a particular type, such as letters, Chinese characters, or words, while the edges represent the relations between these units (Liu, 2008, 2009). Our research focuses on syntactic dependency network in which each vertex represents a word type and each edge the syntactic dependency relation between two words.

The linguistic term *syntax* is used here to describe the principles and processes by which sentences are organized and constructed in particular languages. Phrase structure and dependency structure are two principal methods to analyze syntax. Under the dependency approach, an actual sentence is built out of words linked together by dependencies (Mel'čuk, 1988). Dependency grammar is an approach that arranges syntactic units, i.e., the words, according to the dependency relation (Liu, 2009; Hudson, 2010).

**Figure 1:**
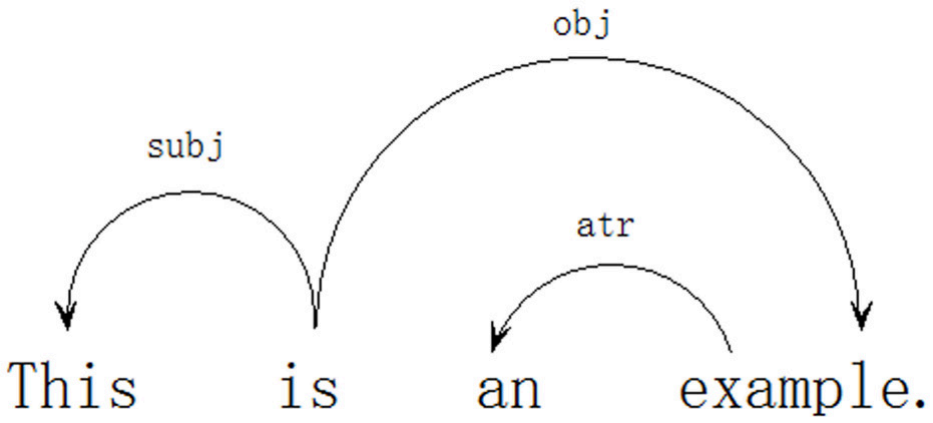
**Dependency analysis of the sentence “*This is an example*.”**.

**Figure 2:**
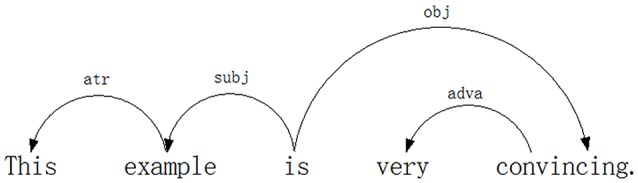
**Dependency analysis of the sentence “*This example is very convincing*.”**.

What drives dependency grammar is simple: all but one word depend on other words. The one word that does not depend on any other words is called the *root*^1^ of the sentence (Debusmann, 2000). Using dependency grammar, two English sentences “*This is an example*” and “*This example is very convincing*” are analyzed in Figures 1, 2.

A syntactic dependency relation is an asymmetric relation between *Dependent* and *Governor*. It can be represented by an arrow (or directed arc) pointing from the governor to the dependent, with the label on the arrow referring to the dependency type. As shown by Figure 1, for instance, *this* is governed by *is, an* is governed by *example*; *example* is governed by *is*; and *is* is not governed by anything (i.e., *is* is the root of the sentence). The one word that does not depend on any other words is called the *root* of the sentence (Debusmann, 2000).

A syntactic dependency **treebank** is constructed on the basis of syntactic dependency analysis of all the sentences in a corpus. Table 1,^2^ is a syntactic dependency treebank based on two English sentences (as shown in Figure 1 and Figure 2) “*This is an example* and *This example is very convincing*.”

**Table 1:** **A syntactic dependency treebank based on two sentences**.

**Order number of sentence**	**Dependent**			**Governor**			**Dependency type**
	**Order number**	**Word**	**POS**	**Order number**	**Word**	**POS**	
1	1	This	pro	2	Is	v	subj
1	2	Is	v				
1	3	An	det	4	Example	n	atr
1	4	Example	n	2	Is	v	obj
2	5	This	pro	6	Example	n	atr
2	6	Example	n	7	Is	v	subj
2	7	Is	v				
2	8	Very	adv	9	Convincing	adj	adva
2	9	Convincing	adj	6	Is	n	obj

**Figure 3:**
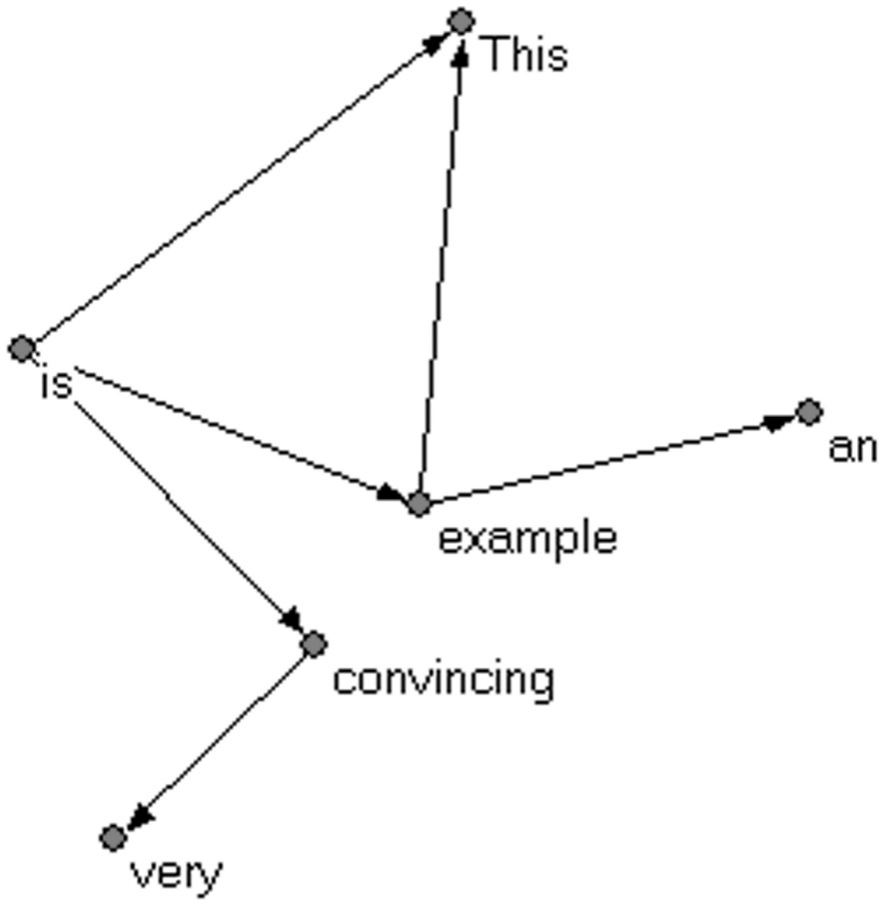
**An example of syntactic dependency network**.

Each row in the table gives a dependency relation that includes three pieces of information: dependent, governor, and dependency type. If a sentence contains *n* words and a complete dependency structure is specified, then it has *n* − 1 dependencies. As a syntactic dependency treebank is a list of all the syntactic dependency relations in a corpus, it can be easily converted into a syntactic dependency network. Using the network analysis software **Pajek**^3^, a syntactic dependency network based on the treebank of Table 1 is drawn (see Figure 3).

The vertices in a syntactic dependency network are actually word types, as opposed to word tokens. The asymmetric dependency relation between two word forms is presented through a directed link between these two vertices. Take the word type *example* as an example: in Table 1, words *This*, and *an*, are governed by the same governor, i.e., *example*; the word *example*, as a dependent, is governed by the word *is*. Thus, there are two edges linking from the vertex *example* to the vertices *This*, and *an*; and one edge linking from the vertex *is* to the vertex *example*, as shown in Figure 3.

### Background information of the DHH and NH students and the corpora construction

We collect samples of writing from both Chinese DHH students and NH peers. The Chinese DHH students come from a school (located in Guangzhou Province, China) for deaf children only. The school have ~400 students distributed among six elementary school grades, three middle school grades, and three high school grades. A questionnaire requesting students' background information was administered to 152 members across 4 grades (from grade 4 to 7). The information covered by the questionnaire is shown in Table 2.

**Table 2:** **Questionnaire concerning the background information of the DHH students**.

** 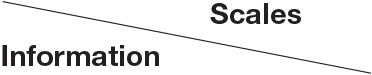 **	**1**	**2**	**3**	**4**	**5**
Other physiological defects	Yes	No			
Age	10–15	Younger than 10	Older than 15		
Level of intelligence	Normal	Abnormal			
Degree of deafness	Moderate	Severe	complete		
Chinese level	Lower	Middle and lower	Middle	Middle and high	High
Both parents are deaf people	Yes	No			
Ways of communication	Sign language	Spoken language	Sign and spoken language		

DHH students appropriate for our comparative study must meet all of the following criteria: (1) they suffer from a severe and complete deafness, but no other physiological defects; (2) their ages range from 10 to 15 years old; (3) they have a normal level of intelligence; (4) their Chinese proficiency is at middle level or above; (5) their parents are both deaf individuals; (6) they only use sign language to communicate. The purposes of these criteria are as follows: (1) To diminish the influences (including other physiological defects except deafness, age differences, different levels of intelligence, different degrees of deafness, and their parents' deafness) that might affect their performance in Chinese writing; (2) To minimize the difference in quality of writing of the DHHs caused by different levels of proficiency; (3) To achieve better contrast between the DHHs (who only use sign language) and the NHs (as spoken language users)^4^. The intermediate DHH students, i.e., who use spoken language or both spoken and sign language to communicate, are excluded from our study. Studies of the written language features of the intermediate DHH students are left for future research. DHH students mainly acquire Chinese written language on Chinese classes. Chinese teachers usually use sign language and exaggerate spoken language to teach. The primary learning styles of Chinese written language for the DHH students are mainly through Chinese *pinyin* and *pinyin* figuring.

These DHH students were required to write compositions with several topics that are appropriate to their age. There are altogether 123 writings collected from 78 DHH students from grade 4 through grade 7. The mean length of their writing is ~171 word tokens. Then these texts were combined as the corpus of the NHHs and a syntactic dependency treebank of this corpus was obtained according to annotation principles of dependency grammar, as shown in Table 1. This dependency treebank contains 21,144 word tokens (~42,200 Chinese characters without punctuations) and its mean sentence length is 17.61 words.

The corpus of the NH peers was based on writing samples selected from students in an ordinary school including six primary grades and three middle grades. This ordinary school is located in the same city as the school for DHH students. Writing samples were selected from the students whose grades range from 4 to 7. The ages of these students range from 10 to 15 years old. Similar to the DHH students, the NH students were required to write compositions with the same topics. Due to the longer mean length of every piece of their writing, 64 pieces of writing were collected from 57 NH students in order to keep a similar size with that of the DHHs' corpora. The syntactic dependency treebank of the NHs contains 20,986 word tokens (~41,000 Chinese characters without punctuations) and its mean sentence length is 16.67 words.

**Figure 4:**
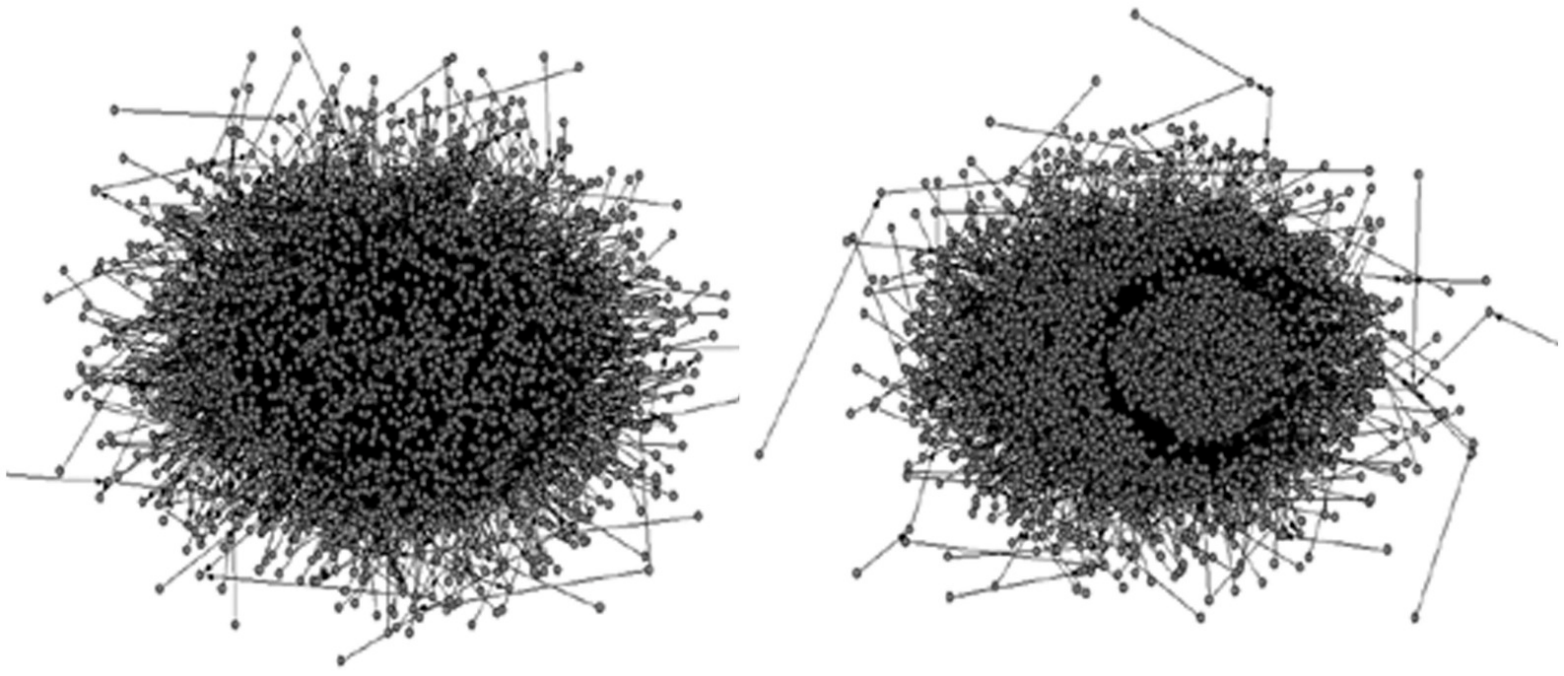
**The syntactic dependency network structures of DHHs Chinese writing (on the left) and their NHs Chinese writing (on the right)**.

The two dependency treebanks were then transformed into syntactic dependency networks using the software **Pajek** (see the transformation procedure in Appendix I in Supplementary Material). They will be called the DHH network and the NH work, respectively hereafter. Their global network graphs (including all the vertices/word types) are presented in Figure 4 (see the drawing process in Appendix I in Supplementary Material).

After we obtained the two syntactic dependency networks, results of relevant network measures were calculated using **Pajek**. Network properties that could provide information about the topological structures of the two networks will be introduced in Section Network Properties.

### Network properties

There is a wide range of quantitative measures (Ferrer-i-Cancho, 2005; Boccaletti et al., 2006; Newman, 2010; Estrada, 2011; Liu, 2011) available for the characterization of the macroscopic properties of linguistic networks, which cover different aspects of the complex organization of relevant language sub-systems (Cong and Liu, 2014). The quantitative analysis of the two syntactic dependency networks can reveal the macro-structures of the syntactic sub-systems of the DHHs and the NHs; and further, discrepancies of writing abilities between the two groups. In our study, we will focus on the following network measures, namely, degree, average degree, clustering coefficient, average path length, small-world and scale-free structures, and network centralities.

#### Degree

In a network model, a vertex, unless it is an isolated one, will link to another vertex or to other vertices. A vertex's ***degree*** refers to the number of edges that connect to it, which manifests the connectivity of that vertex. In a directed network, a vertex has both in-degree (the number of ingoing edges) and out-degree (the number of outgoing edges). Take the syntactic dependency network in Figure 3 as an example, the in-degree and out-degree of the vertex “*convincing*” are both 1. In a syntactic dependency network, the degree of any given vertex is an estimate of all the possible syntactic dependency relations it can form with other words, and thus a measure for the corresponding word's combinatorial capacity to form syntactic dependency relations, i.e., its *syntactic valency* (Cong and Liu, 2014).

***The average degree <k>*** of a network is the mean of all its nodes' degrees. By using the software **Pajek**, the average degree of the syntactic dependency network in Figure 3 can be calculated as 2.0. The calculating procedure is displayed in Appendix I in Supplementary Material.

#### Clustering coefficient

In graph theory, the clustering coefficient is a measure of the degree to which vertices in a graph tend to cluster together. It is a measure of transitivity in a network. In a linguistic network, the neighbors of a given linguistic unit (as a vertex) may be neighbors themselves. This tendency is measured by a probability called the ***clustering coefficient*** of the linguistic unit as a network vertex (Newman, 2010).

Clustering coefficient *C*_*i*_ of a vertex *i* is the ratio of the total number *E*_*i*_ of edges that actually exist between all its *k*_*i*_ nearest neighbors and the number *k*_*i*_(*k*_*i*_ − 1)*/*2 of all possible edges between them, i.e.,

(1) $$\begin{array}{l} C_{i}=\frac{2E_{i}}{k_{i}\left(k_{i}-1\right)} \end{array}$$

The clustering coefficient *C* of the whole network is the average of all individual *C*_*i*_, which is formulated as:

(2) $$\begin{array}{l} C=\frac{1}{N}\overset{N}{\underset{i=1}{\sum }}C_{i} \end{array}$$

Loops are deleted before calculating the clustering coefficient *C*. The clustering coefficient *C* of the whole network is calculated using **Pajek** and the calculation process is shown in Appendix I in Supplementary Material.

#### The average path length

The ***average path length (L)*** of a network is defined as the shorted path length averaged over all possible pairs of vertices.

(3) $$\begin{array}{l} L=\frac{1}{\frac{1}{2}N\left(N-1\right)}\sum_{i>j}d_{ij} \end{array}$$

In this formula, *N* is the number of vertices in the network; *d*_*ij*_ indicates the distance, or the length of the shortest path between vertex *i* and vertex *j*. *d*_*ij*_ can be defined as the number of edges in a shortest path that linking two vertices. The average path length of the syntactic dependency network can be calculated by the software **Pajek**, as shown in Appendix I in Supplementary Material. If there are more than one components in a network graph, then the value of *L* is calculated based on the largest component of that network.

#### Scale-free and small-world network structures

The degree of a vertex in a network is the number of edges on (i.e., connected to) that vertex. We then define *P*(*k*) to be the fraction of vertices in the network that have degree *k*. Equivalently, *P*(*k*) is the probability that a vertex chosen uniformly at random has degree *k*. Thus, *P*(*k*) can be seen as a function of degree *k*. This function is the ***degree distribution*** for the network. In a random graph, the degree distribution follows binomial distribution or Poisson distribution (as the limit of binomial distribution in large graphs; Newman, 2003, 2010). The ***random network*** in our research, with connections placed among the vertices at random, is assuming a version that satisfies two requirements: (1) the number of edges and the number of vertices are the same as those in the original graph; and (2) all pairs of vertices have the same probability of being connected, which leads to a binomial distribution.

If the degree distribution of a network generally follows a power law distribution, then network of this type is generally a scale-free network (Newman, 2003, 2005, 2010; Ferrer-i-Cancho et al., 2004; Clauset et al., 2009). Different from binomial or Poisson distribution, the degree of the vertices in scale-free networks are highly right-skewed, meaning that there are many vertices with few connections and a small number of vertices with many connections. This power-law distribution can be described by,

(4) $$\begin{array}{l} P\left(k\right)\sim k^{-\gamma } \end{array}$$

The small-world organization of a complex network is determined by two measures (Ferrer-i-Cancho and Solé, 2001; Newman, 2003, 2010; Ferrer-i-Cancho et al., 2004; Newman et al., 2006; Cong and Liu, 2014). One is the average path length (*L*), and the other is the clustering coefficient (*C*). A network is a small-world network if (1) ***the average path length*** *L* is almost as small as that of its random network counterpart, and (2) ***the average clustering coefficient*** is far greater than that of its random network counterpart.

Real-world networks, such as biological, social, and technological ones are complex networks (Dorogovtsev et al., 2008; Newman, 2010). Many systems in nature are composed of a large number of interacting components that exhibit scale-free, small-world, and hierarchical behavior (Caldarelli, 2007). Similarly, human language is a dynamic, self-organizing complex system (Dorogovtsev et al., 2008; Liu and Cong, 2014), with small-world and scale-free structures being the most popular features (Ferrer-i-Cancho and Solé, 2001; Ferrer-i-Cancho et al., 2004; Masucci and Rodgers, 2006; Liu, 2008; Liu and Cong, 2014). Whether the syntactic dependency networks of the DHH and NH students also display small-world and scale-free structures is worth exploring. Relevant analysis will be presented in Results Section Scale-Free and Small-World Structures of the Two Networks.

### Network centralities

**Figure 5:**
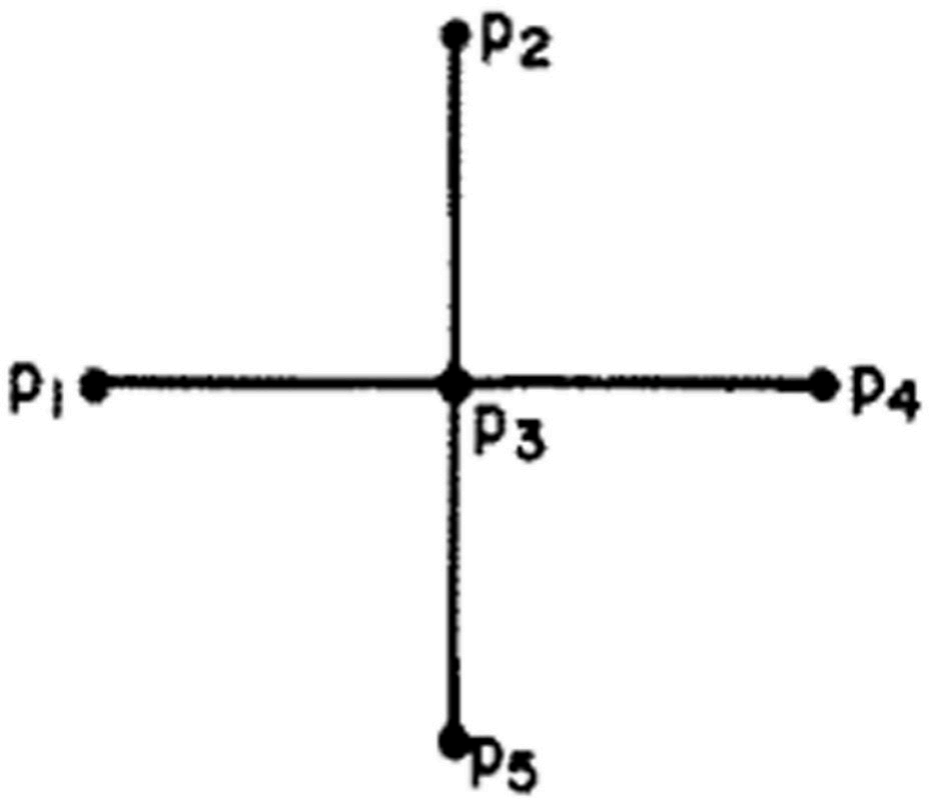
**A simple graph with five vertices**.

Centrality is another important quantitative measure for the characterization of the topological properties of syntactic dependency networks. A vertex located in the center of a star-like graph (as shown in Figure 5) is widely assumed to be structurally more central and thus more important than any other vertices in any other position in the graph. Previous research (Freeman, 1979; Wasserman and Faust, 1994) attempted to grapple with the ways in determining the structural uniqueness of such a central position. It turns out that there are three distinct structural properties that are uniquely possessed by a central vertex: the vertex has the maximum possible *degree*; it falls on the geodesics *between* the largest possible numbers of other vertices and, since it is located at the minimum distance from all other vertices, it is maximally *close* to them (Freeman, 1979). We thus use the three network centrality measures, i.e., ***degree***, ***betweenness centrality***, and ***closeness centrality*** to describe the global importance of a vertex.

As introduced in Section Degree, a vertex's ***degree*** refers to the number of edges that connect to it. The definition of the other two centrality measures are introduced as follows.

In the field of social science, a point (vertex) in a communication network is central to the extent that it falls on the shortest path between pairs of other points (Freeman, 1977). This same intuition was expressed by Shimbel (1953). Such is the consideration underlying the concept of ***betweenness centrality***. It is computed only for networks that do not contain multiple edges. It is equal to the number of shortest paths from all vertices to all others that pass through that node. The betweenness centrality of a vertex *v* is given by,

(5) $$\begin{array}{l} C_{B}\left(v\right)=\sum_{i\neq j}\frac{G_{v}\left(i,j\right)}{G\left(i,j\right)} \end{array}$$

where *G*_*v*_(*i, j*) is the number of shortest pathways between *i* and *j* running through *v* and *G*(*i, j*) = ∑_*v*_*G*_*v*_(*i, j*) (Ferrer-i-Cancho et al., 2004).

In a network graph, there is a natural distance metric between all pairs of vertices, defined by the length of their shortest paths. The farness of a vertex *v* is defined as the sum of its distances to all other vertices, and its closeness is defined as the reciprocal of its farness (Bavelas, 1950; Sabidussi, 1966). That being so, the more central a vertex is, the lower its total distance to all other vertices, and thus it is considered more important in that network. Generally speaking, the definition of ***closeness centrality*** is that it is the reciprocal of the total distance from a particular vertex to all other vertices,

(6) $$\begin{array}{l} C_{C}\left(v\right)=\frac{n-1}{\sum_{j\neq i}L_{ij}} \end{array}$$

Both betweenness centrality and closeness centrality of syntactic dependency networks are calculated using **Pajek**. Detailed calculation processes are presented in Appendix I in Supplementary Material. In the following sections, we will provide the results of those network measures, followed by their corresponding discussions and relevant implications.

## Results

### Scale-free and small-world structures of the two networks

First, we will discuss whether these two syntactic dependency networks exhibit scale-free and small-world structures, for most linguistic networks of human language (Ferrer-i-Cancho and Solé, 2001; Ferrer-i-Cancho et al., 2005; Markošová, 2008; Biemann, 2012) have the same features.

**Figure 6:**
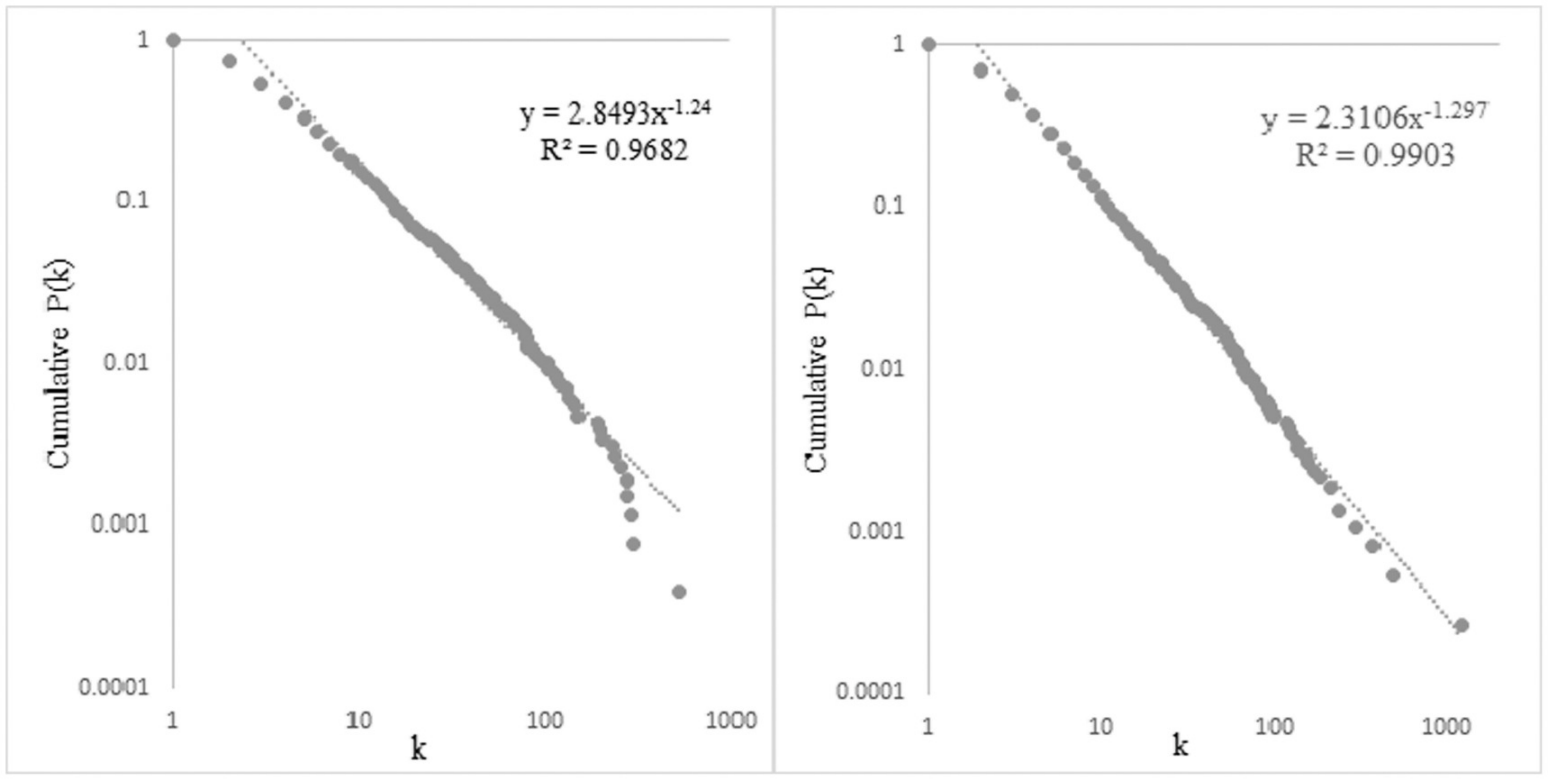
**The cumulative degree distributions of the syntactic dependency networks of the DHHs (left) and the NHs (right)**.

**Figure 7:**
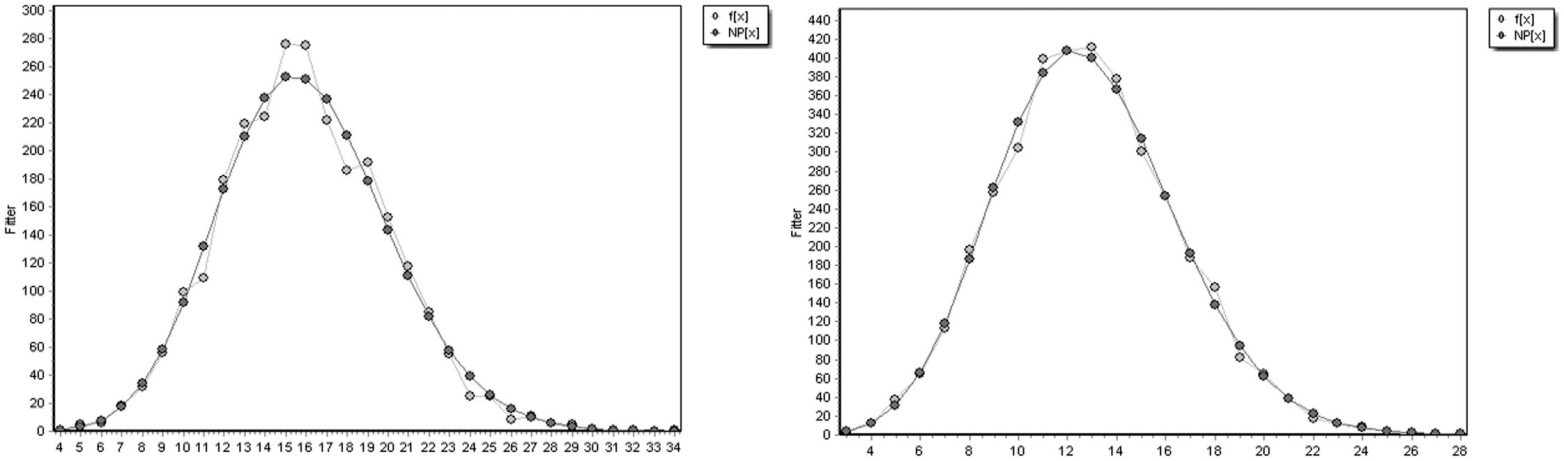
**The degree distributions of the random networks of the DHH (left) and the NH (right)**.

As introduced in Section Scale-Free and Small-World Network Structures, on the one hand, the cumulative degree distributions of both original networks and their random networks need to be observed for a scale-free network structure (Newman, 2003; Clauset et al., 2009). The cumulative degree distributions (in log-log scales) of the two networks are shown in Figure 6. The degree distributions of the two corresponding random networks are shown in Figure 7.

It can be observed in Figure 6 that both syntactic dependency networks display a power-law-like distribution. Their cumulative degree distributions are fitted by a linear power law with the slope of −1.24 for the DHH network, and −1.297 for the NH network. The determination coefficient *R*^2^ is 0.9682 for the DHH network; and *R*^2^ is 0.9903 for the NH network. The greater value of *R*^2^ for the NH network quantitatively suggests that the network of NH peers is more power-law-like. A greater deviation from a power-law at the right end of the distribution for the DHH network helps to interpret the higher *R*^2^ of the NH network. In Figure 7, different from the power-law degree distributions of the original networks, both random networks display a binomial like distribution, with *R*^2^ = 0.987 for the DHH network, and *R*^2^ = 0.996 for the NH network. The power-law-like degree distributions in both original networks suggest that, both networks exhibit a scale-free structure. Moreover, the greater value of *R*^2^ for the NH network and the greater deviation from a power-law at the end of the distribution for the DHH network indicate that the NH network displays a more power-like degree distribution.

On the other hand, we calculated the values of the average path length and the clustering coefficient of both networks and their corresponding random networks. The statistical results are displayed in Table 3.

**Table 3:** **Major measures of the two networks and those of their corresponding random networks**.

**Networks**	**Word tokens**	***N***	***<k>***	***C***	***L***
DHH	21,144	2592	7.927	0.135	3.983
Random-DHH	21,144	2592	7.927	0.003	4.027
NH	20,986	3711	6.437	0.117	3.815
Random-NH	20,986	3711	6.437	0.002	4.626

*N, the number of vertices; <k>, average degree; C, clustering coefficient; L, average path length*.

Recall that the distinctive combination of high clustering coefficient with short average path length characterizes the small-world structure of a network. As the results shown in Table 3, the values of C of the two networks are far greater than those of their corresponding random networks, whereas their values of *L* almost as small as those of the latter. These results suggest that both networks display a small-world structure. In addition, it is known that the value of the average path length *L* is expected to be smaller in a network with a power-law-like degree distribution (Cohen and Havlin, 2003). As shown in Table 3, *L*_*DHH*_ < *L*_*random-DHH*_, and *L*_*NH*_ < *L*_*random-NH*_. These results are consistent with our first relevant analysis that both original networks are scale-free.

A finite *L* needs that the graph is connected. There are three components in the DHH network, and only one component in the NH network (indicating a connected graph). More components in the DHH network indicates that it is less connected. Reasons for the less connectedness of the DHH network are left for future research. We then extracted the largest connected component in the DHH network. This largest connected component in the DHH network has 2589 vertices, which constitutes 99.984% of the whole network. The software **Pajek** is using the *L* of the largest connected component to represent the value of *L* of the whole network. Since the DHH network has three components, and the largest connected component almost covered all the vertices and edges of the original network, whereas the other two components are too small to be the representatives of the whole network, therefore we use *L*_*largest*_ to represent the *L* of the whole DHH network in our study.

### Comparison of network properties of the DHH and NH networks

Table 3 provides the main measures, i.e., the number of vertices, the average degree, and clustering coefficient, and the average path length (*L*), of two networks for comparison. A non-parametric Mann-Whitney *U*-test was conducted to explore whether there are significant differences between the two networks in these measures. The result of the test suggested that the two network exhibit significant differences in three network measures (*p* < 0.001 for the clustering coefficient, *p* = 0.002 for the average degree, and *p* = 0.001 for average path length). Further, discussions will be presented in Section Comparisons of the Network Properties between the Two Networks.

### Network centralities and function words

The centrality indices of function words in the two syntactic dependency networks are compared. There are two reasons why function words are examined here. First, according to the definitions of centrality indices in Section Network Centralities, all of them are described as indices of frequency, prestige, prominence, importance, and power of the vertices from a global point of view (Freeman, 1979). In a syntactic dependency network, centrality measures may reflect the relative combinatorial capacity of word types (Liu and Cong, 2014). The higher the value of a vertex's centrality indices, the greater the relative strength of that vertex behaving as hubs. The vertices with extremely high network centrality, i.e., the hubs, tend to be function words (e.g., articles and prepositions, etc.; Ferrer-i-Cancho and Solé, 2001; Solé et al., 2010; Chen and Liu, 2011; Cong and Liu, 2014). These results imply that function words are probably in important central positions in syntactic networks. Second, Chinese is considered as an analytic language, relying on word order and particles (i.e., function words^5^), instead of inflection or affixes, to construct syntactic or grammatical patterns (Norman, 1988). Function words have little lexical meaning or have ambiguous meaning, serving to express grammatical relationships with other words, or to specify the attitude or mood of the speaker. They signal the structural relationships that words have to one another and they are the glue that holds sentences together. Thus, they serve as important elements to the structure of sentences (Klammer et al., 2010). The use of function words by Chinese language learners can reflect their grammatical or syntactic ability to some degree. Network centrality measures provide a good opportunity to observe the usage of function words in syntactic dependency networks.

First, there are altogether 3539 function words (word tokens) in the DHH corpus, and 4824 function words (word tokens) in the NH corpus. The total number of words (word tokens) of the DHH corpus is 21,141, and 20,986 for the NH corpus. Then it is easy to calculate the actual percentages of the use of function words in both corpora using the following formula,

(7) $$\begin{array}{l} P_{f}=\frac{n_{f}}{N}\times 100\% \end{array}$$

where *n*_*f*_ is the total number of function word tokens, and *N* is the total number of word tokens. *P*_*f*_ is 12% for the DHH corpus, and 23% for the NH corpus. Chi-square test shows that the difference in the actual percentage (12% vs. 23%) of the use of function words is statistically significant, with *p* < 0.001. This result quantitatively reveals that the DHH students use less function words than the NH peers in their writing.

Second, there are 128 function word types in both syntactic dependency networks. Space precludes a complete list with every function word. Thus, only 20 function words together with their three centrality measures are presented in Tables 4–6, respectively. In the three Tables: “c” is short for conjunction; “d” is short for adverb; “p” is for preposition; “pbei” refers to a special Chinese character of preposition, indicating passive tense; “udel,” a relative marker, refers to Chinese character “的,” which is similar to the meaning and usage of “*of*” in English.

**Table 4:** **^6^Degree of 20 function words in the two networks^7^**.

**Network of DHH students**			**Network of NH students**		
**Degree**	**Function words**	**Part of speech**	**Degree**	**Function words**	**Part of speech**
529	的	ude1	1231	的	ude1
202	在	p	211	在	p
230	和	c	126	和	c
78	不	d	89	不	d
7	与	p	78	与	p
46	就	d	77	就	d
92	给	p	71	给	p
46	又	d	66	又	d
44	对	p	66	对	p
39	也	d	63	也	d
17	为	p	62	为	p
32	都	d	61	都	d
35	用	p	55	用	p
23	被	pbei	48	被	pbei
9	就是	d	47	就是	d
17	向	p	43	向	p
15	从	p	35	从	p
7	当	p	35	当	p
4	而	c	33	而	c
20	只	d	31	只	d

**Table 5:** **Betweenness centrality (Bc) of 20 function words in the two networks**.

**Network of DHH students**			**Network of NH students**		
**Bc**	**Function words**	**Part of speech**	**Bc**	**Function words**	**Part of speech**
0.1880	的	ude1	0.3105	的	ude1
0.0360	在	p	0.0256	在	p
0.0259	和	c	0.0112	和	c
0.0082	给	p	0.0069	给	p
0.0004	与	p	0.0055	与	p
0.0043	对	p	0.0053	对	p
0.0017	对	p	0.0051	对	p
0.0026	用	p	0.0039	用	p
0.0001	就是	d	0.0023	就是	d
0.0000	当	p	0.0015	当	p
0.0010	从	p	0.0013	从	p
0.0011	于	p	0.0012	于	p
0.0006	向	p	0.0011	向	p
0.0000	才	d	0.0010	才	d
0.0000	以	p	0.0010	以	p
0.0000	因	p	0.0009	因	p
0.0000	只有	c	0.0009	只有	c
0.0000	别	d	0.0008	别	d
0.0000	而	c	0.0006	而	c
0.0000	或	c	0.0006	或	c

**Table 6:** **Closeness centrality (Cc) of 20 function words in the two networks**.

**Network of DHH students**			**Network of NH students**		
**Cc**	**Function words**	**Part of speech**	**Cc**	**Function words**	**Part of speech**
0.4875	的	ude1	0.5354	的	ude1
0.4473	在	p	0.4361	在	p
0.4472	和	c	0.4161	和	c
0.4140	给	p	0.4090	给	p
0.3942	对	p	0.4059	对	p
0.3165	就是	d	0.3995	就是	d
0.2985	与	c	0.3915	与	c
0.3152	而	c	0.3871	而	c
0.3556	为	p	0.3867	为	p
0.3821	就	d	0.3831	就	d
0.3901	不	d	0.3817	不	d
0.3287	才	d	0.3805	才	d
0.3786	真	d	0.3752	真	d
0.3773	都	d	0.3690	都	d
0.3444	经过	d	0.3690	经过	d
0.3878	也	p	0.3677	也	p
0.3156	当	d	0.3644	当	d
0.2808	只有	d	0.3605	只有	d
0.2498	不行	d	0.3580	不行	d
0.2763	渐渐	d	0.3573	渐渐	d

For the same 128 function words in both networks, we calculated their average degree (DHH = 19.180, NH = 23.250), the mean value of their betweenness centrality (DHH = 0.003, NH = 0.002), the mean value of their closeness centrality (DHH = 0.317, NH = 0.378). Results of non-parametric Kolmogorov-Smirnov test suggested that, the three centrality indices of function words of DHH students are significantly lower than those of NH peers (*p* < 0.001 for the degree, *p* = 0.001 for the betweenness centrality, and *p* < 0.001 for the closeness centrality).

## Discussions

### Small-world and scale-free structures of the two syntactic dependency networks

Previous research suggests that writing of teenagers is still in a developing stage before they reach language maturity (Hudson, 2009). The language immaturity of language learners can be manifested in various linguistic aspects, such as vocabulary size, mean sentence length, syntactic complexity, etc. (Brown, 1973; Shore, 1994). Similar to other natural human languages (Ferrer-i-Cancho and Solé, 2001; Ferrer-i-Cancho et al., 2004, 2005; Ferrer-i-Cancho, 2005; Dorogovtsev et al., 2008; Cong and Liu, 2014), our results show that both DHH and NH networks are also small-world and scale-free.

Small-world networks are identified by their shortest path lengths and clustering coefficient (Ferrer-i-Cancho and Solé, 2001; Newman, 2003, 2010; Vitevitch, 2008). A small average path length enables rapid transmission of message through the network and thus affords the network great processing efficiency (Vitevitch, 2008). If a word is reached in communication, jumping to another word requires only very few steps (Ferrer-i-Cancho and Solé, 2001). A cluster means that two neighbors of a given vertices are also connected to each other as well (Newman, 2003). Small-world networks have a high clustering coefficient, indicating that the neighbors of a given vertex are highly interconnected (Vitevitch, 2008). This topological feature implies a robust network which is less easily to be destroyed (Ferrer-i-Cancho and Solé, 2001; Vitevitch, 2008). In addition, such topological features of linguistic networks help to facilitate communication between vertices, and thus facilitates the navigation (Cong and Liu, 2014).

The distinguishing feature of scale-free networks is their power-law-like degree distribution. A power-law-like degree distribution suggests that a small number of vertices have extremely high degrees while most vertices have rather low degrees. The scale-free property of a linguistic sub-system can be interpreted as the heterogeneity of the distribution of the word types' combinatorial capacity (Liu, 2009; Cong and Liu, 2014). As Figure 6 shows, though both DHH and NH networks display a power-law-like degree distribution, there are still differences at the right end. The degree distribution of the NH network is more power-law-like. In Figure 6, those dots at the end of both lines are actually representatives of the word types that are having extremely high degrees. A more power-law-like degree distribution at the end of the curve of the NH network suggests that, NH students have a better command of lexical use and lexical collocations. This fact also implies a higher syntactic proficiency level for the NH students.

Complex network structures could help to understand the organization and dynamics of cognitive and behavioral process of human brain (Baronchelli et al., 2013; Boersma et al., 2013). The similar small world feature of the syntactic sub-systems between the DHHs and the NHs suggests a similar dynamic procedure in constructing syntactic patterns to some degree. However, more studies need to be conducted concerning this hypothesis. Our finding of the small-world and scale-free features of the two networks can be a complement to the general features of human language.

### Comparisons of the network properties between the two networks

According to the results in Section Comparison of Network Properties of the DHH and NH Networks, the differences of these statistical measures between the two networks and relevant possible cognitive and linguistic implications will be discussed as follows.

First, recall that the vertices of a syntactic dependency network are the word types, as opposed to the word tokens. Table 3 shows that there are 21,144 word tokens in the DHH corpus, and 20,986 word tokens in the NH corpus; in terms of the word types (i.e., the number of network vertices), there are 2592 word types in the DHH network, and 3711 in the NH network. The Type/Token ratios (TTR) have been extensively used in child language research as an index of *lexical diversity/vocabulary richness* (Richards, 1987). The TTR for the DHHs is 12%, and 18% for the NHs. Therefore, a larger lexical diversity or a higher vocabulary richness can be found in the NH writing.

The second analysis concerns the clustering coefficient. According to the results in Section Comparison of Network Properties of the DHH and NH Networks, the clustering coefficient of the DHH network is 0.135. It is significantly higher than that of the NH network, i.e., 0.117. Recall that the DHH network has three components, and the NH network has a single component. The less connectedness may be one of the distinguishing features of the DHH network. The largest connected component of the DHH network constitutes 99.984% of the whole network, thus we use the clustering coefficient *C* of the largest connected component to represent the *C* of the original DHH network^8^.

Previous research suggested relative correlations exist between the network property of clustering and language acquisition or language processing (Charles-Luce and Luce, 1990, 1995; Vitevitch, 2006, 2008; Lerner and Ogrocki, 2009; Beckage et al., 2011; Goldstein and Vitevitch, 2014). Vitecitch and his colleagues made abundant research on the cognitive costs associated with clustering coefficients (Vitevitch, 2006, 2008; Goldstein and Vitevitch, 2014). They found out that words with a high clustering coefficient were responded more quickly than words with a low clustering coefficient (Vitevitch, 2006). They also argued that words with a low clustering coefficient were more easily acquired or learned than words with high clustering coefficient (Vitevitch, 2008). Charles-Luce and Luce (1990, 1995) found out that the neighborhood density for words in the adult lexicon was greater than that for those same words in the 5 and 7 years old lexicon. Beckage et al. (2011) also suggested a decreased clustering in associative networks of late talkers. It seems that a higher neighborhood density was found in language users with higher linguistic competence, which is interestingly inconsistent with our finding. Interestingly, a rise of clustering in word fluency networks was found in Alzheimer patients (Lerner and Ogrocki, 2009). And this finding is somewhat similar to ours, i.e., a significant higher clustering for the DHH network. It is unclear whether the inadequate linguistic input for deaf individuals may lead to the similar clustering topology with Alzheimer patients pathologically. Different linguistic sub-systems network models (such as word-occurrence network, syntactic network, or syntactic network) may also lead to different observations. More psychological studies are needed on the correlation between network clustering and language processing.

Here, we provide a simple argument for the higher clustering of the DHH network by using a null hypothesis, i.e., a random binomial graph of the original network^9^. A random binomial graph/network is assuming a version that satisfies two requirements: (1) the number of edges and the number of vertices are the same as those in the original network; (2) all pairs of vertices have the same probability of being connected, which leads to a binomial distribution. If there are *M* edges and *N* vertices in a binomial random graph, the probability that any two vertices are neighbors is *p* = *2M/N*(*N* − *1*); moreover, the average degree *c* in a binomial graph with *M* edges and *N* vertices is equal to *c* = *2M/N*, thus we have *p* = *c/*(*N* − 1) (assuming that loops are not allowed). The clustering coefficient *C* is defined as the probability that two network neighbors of a vertex are also neighbors of each other. *C* is approximately *p* in a random binomial graph, hence *C* = *c/*(*N* − 1) (Newman, 2010). While *c* (the average degree) is about the same in the DHH and NH networks, *N* is much larger for NH. It implies that (under the null hypothesis) the clustering coefficient is expected to be smaller in the NH network. Sophisticated arguments, however, can be made assuming a power-law distribution, and this could be left for future research.

The third analysis involves the statistical measure of the average degree. Our results indicate that the average degree of the DHH network is significantly lower than that of the NH network. Figure 6 shows that both networks are scale-free (i.e., having power-law-like degree distributions). It implies only a small number of vertices have extremely high degrees while most vertices have rather low degrees. A small number of vertices with high degrees usually behave as ***hubs*** of their network (Barrat et al., 2008; Cong and Liu, 2014). The lower value of the NH average degree is probably due to a higher degree of their lexical diversity (i.e., a larger number of their word types), since most word types (i.e., network vertices) have rather low degrees. Accordingly, the average degree of the NH network is reduced.

The following analysis is the statistical measure of the average path length (*L*). Our results suggest that the shortest path length for the DHH network is significantly higher than that of the NH network. Nishikawa et al. (2002) discuss that the average path length *L* of a small-world network is the smallest when all paths are connected only to a single center node. Other studies also suggest that hubs play an important role in the reduction of the level of *L* of a network (Watts and Strogatz, 1998; Nishikawa et al., 2002). In syntactic dependency networks, vertices with extremely high degrees, i.e., the hubs, tend to be function words (e.g., articles and prepositions), (Solé et al., 2010; Cong and Liu, 2014). Chinese as an analytic language relies on word order and particles (i.e., function words), instead of inflections or affixes, to form grammatical patterns, such as person, number, tense, mood, or case (Norman, 1988). Function words play an important role in reducing the average path length (*L*) of its syntactic dependency network. Therefore, the *L* of the DHH network tends to be lower than that of the NH network, for the latter is more reliant on function words. The lower value of *L* for the DHH network can also imply a lower language proficiency level of writing in their use of function words.

The above analysis suggests that the two networks display significant differences in the main network measures. Network theory offers new perspectives for understanding cognitive complexity (Baronchelli et al., 2013). It provides new insights in understanding the organization and dynamics of cognitive and behavioral process of human brain (Baronchelli et al., 2013). Previous studies (Antiqueira et al., 2007; Amancio et al., 2011; Mota et al., 2014) suggest that various network measures, such as degree, clustering coefficient, the average path length, etc. can be used to quantify the level of complexity by capturing different topological structures of networks. Thus, the significant differences in various network properties could also suggest different levels of complexity of the two syntactic sub-systems, and further, different levels of cognitive complexity in the syntax between the two groups. It is worthy of investigation whether the inadequacy of linguistic input in the early age of DHH students constitutes one of the reasons for the observed differences.

Function words play an important role in constructing complex Chinese sentence patterns (Chen and Liu, 2011). The use of function words is a significant representation of the syntactic ability for Chinese learners. Though the average path length (*L*) provides relative information in terms of function words, more specific research concerning this aspect is still worth studying. Section Network Centralities and Function Words will present a deeper analysis.

### Network centralities and function words

Results in Section Network Centralities and Function Words indicate that the Chinese character “的 (de, a relative marker)” ranks the first in terms of the three network centrality measures. This result reinforces the conclusion that the function word “的(de)” plays the most vital role of network hub in Chinese syntactic dependency networks (Chen and Liu, 2011), indicating that “的(de)” has the highest combinatorial capacity in Chinese syntax. Function words usually behave as the central hubs in syntactic networks (Ferrer-i-Cancho and Solé, 2001; Ferrer-i-Cancho et al., 2004; Chung and Pennebaker, 2007; Solé et al., 2010; Chen and Liu, 2011; Baronchelli et al., 2013), and the positions of hubs are determined by the global structure of the network (Ke and Yao, 2006).

Recall the results displayed in Section Network Centralities and Function Words. First, the ratios of function words are different, i.e., it is 12% for the DHH network, and 23% for the NH network. The numerical discrepancy suggests that, the DHH students are less reliant on function words than the NH peers. Second, the network centrality indices for the same 128 function words in both corpora exhibit significant differences, with the values of the DHH network significantly lower than the values of the NH network. This result implies that DHH students and NH peers have discrepancy in the use of function words in terms of quality.

A number of studies indicate that Chinese language learners have great difficulties in the recognition and the use of function words (Li, 2002; Hicks, 2006; Lee and Chen, 2009; Ma et al., 2009). Modern Chinese studies suggest that function words are of great significance in producing smooth and coherent Chinese sentences and texts. Thus, the syntactic ability of Chinese might be reflected through the use of function words. Moreover, network centrality indices reflect the relative importance of a vertex in a network globally (Freeman, 1979; Wasserman and Faust, 1994). We can speculate that, the higher the value of centrality indices of a vertex, the more important the vertex, and more complex syntactic structures may be accomplished by that vertex (i.e., the word type). The significant lower values of the three centralities for the DHH network indicate that, function words used by the DHHs play a weaker role in the construction of syntactic structures than those used by the NHs. These lower values could be a consequence of the fact that the DHH students have a lower syntactic ability. This lower syntactic ability further indicates less complexity of sentence patterns, less coherent and cohesive sentences or even texts in the DHH writing.

Reasons for the rigid use of function words of DHH students can be various. Researchers may immediately argue that the lack of linguistic input in the early stage of DHH students is possibly the most important and direct reason (Saville-Troike, 2012). In addition, DHH students cannot communicate verbally (including both deaf and normal-hearing) as conveniently and fluently as hearing students. Sign language is a primary means of communication for most deaf students (Dizeu and Caporali, 2005; Staden et al., 2011; Clark et al., 2014). The sign language we refer to here is used for interaction by deaf populations as spoken language is used by hearing population (Stokoe, 2003). It is suggested that in Chinese sign language, the expression of function words were not through signs, but rather through body gestures, or facial expressions, etc. (Wu, 2005, 2006). Therefore, this is another reason for the rigid use of function words of the DHH students.

To conclude, the rigid use of function words for the DHH students is reflected in two aspects: (1) DHH students are less likely to use function words in their writing than the NH peers. (2) DHH students have a lower proficiency level for the use of the same 128 function. Though similar indications might have been found in previous studies, complex network approach provides a macroscopic observation with precise network measures.

Observed from a dependency-based theory of syntax, the two research questions put forward in Introduction can be approached from the following aspects. First, both DHH and NH syntactic networks display small-world and scale-free structures. These network features are consistent with the macroscopic network structures of other natural languages; furthermore, the difference at both ends of the distribution lines suggests a more power-law-like degree distribution for the NH network. Second, the second question is answered from two aspects: on the one hand, the two networks present significant statistical differences concerning the main network measures, namely, the clustering coefficient, the average path length, and the average degree. Discrepancy of their lexical diversity/vocabulary richness and the rigid use of function words of the DHH students may help to explain the observed differences to some degree. On the other hand, the significant statistical differences between the three network centrality measures, i.e., degree, betweenness centrality and closeness centrality, further suggest a rigid use of function words and a lower syntactic ability for the DHH students.

Research with the complex network approach is relatively young, but many important and interesting insights in various aspects of the nature and the human society have already been attained. Research on the complex network structures of human language not only helps to deepen our insights into both the macro- and micro-structures of human language, but also change our understanding in the organization and dynamics of cognitive and behavioral process. Our research on the complex features of the syntactic sub-system model of DHH Chinese writing is a complement to the general features of human language. However, limitations do exist when using the network approach; the network approach can only be used as a tool rather than as the research goal when the purpose is to understand human language. Apart from lexical and syntactic discrepancies between the two groups, other linguistic features, such as phonological, semantic, or pragmatic aspects are not accounted for in our research. More detailed research is necessary to reveal the underlying causes of these discrepancies. Apart from syntactic dependency networks, other linguistic networks (such as a dynamic semantic network, a word-formation co-occurrence network as well as a character co-occurrence network) could also be used to uncover the language features of DHH students. Moreover, these complex network approaches can also be applied when analyzing the sign language of DHH individuals. In addition, because learning to write for deaf signers is essentially the same as learning a second language, so comparison group of non-native speakers of Chinese is another interesting study left for future research.

## Author contributions

Conceptualization: HL and HJ. Investigation: HJ. Data analysis: HJ and HL. Visualization: HJ. Writing—original draft: HJ. Writing—review and editing: HJ and HL. Funding acquisition: HL.

## Funding

This work was supported by the National Social Science Foundation of China under Grant 11&ZD 188.

### Conflict of interest statement

The authors declare that the research was conducted in the absence of any commercial or financial relationships that could be construed as a potential conflict of interest.

## Footnotes

^1^The root is alternatively termed the main or central element.

^2^*pro* is a pronoun, *v* is a verb, *det* is a determiner, *n* is a noun, *adv* is an adverb, *adj* is an adjective, *subj* is subject, *atr* is attribute, *obj* is object, *adva* is adverbial. In addition, all the punctuations have been deleted.

^3^**Pajek**, a program for large network analysis, can be freely downloaded on the Internet. The webpage is http://vlado.fmf.uni-lj.si/pub/networks/pajek/.

^4^DHH students can choose to SPEAK or SIGN or both to communicate, which is largely due to their different language-learning environment of their birth family. Different language-learning environment will profoundly influence their linguistic experiences, which have different cascading effects in language-related areas of development (Meier, 1991; Evans, 2004; Lederberg et al., 2013), such as theory of mind, conceptual skills, reading strategies, and writing composition skills, etc. Therefore, an ambiguous selection of the DHH students will lead to a confusing result of our study. Thus the DHH students who use spoken language to communicate are excluded from our study.

^5^It is generally accepted that adverbs, conjunctions, prepositions, particles, and interjections are considered to be function words in Chinese traditional grammar.

^6^English counterparts of these Chinese function words in Tables 4–6 are presented in Appendix II in Supplementary Material.

^7^Each table displays the top 20 function word types in terms of one centrality measure in the NH network, together with their values of this centrality measures in both networks.

^8^We should notice that, though the largest connected component constitutes 99.984% of the original network, the extraction of the largest *C* may still cause some statistical biases.

^9^This argument for the higher clustering of the DHH network was suggested by the reviewer.
