# Supplementary material for: Chinese Writing of Deaf or Hard-of-Hearing Students and Normal-Hearing Peers from Complex Network Approach
Source: Front Psychol. 2016 Nov 22;7:1777. doi: 10.3389/fpsyg.2016.01777 (PMC5119054; doi:10.3389/fpsyg.2016.01777)
Supplement: Supplementary file 1 [file DataSheet1.docx]

**Appendix I**

With the syntactic dependency network in **Figure 3** as an example, we list the overall procedure for the analysis of a syntactic dependency network as follows:

1. Treebank building. Our treebank takes the form of an Excel table (see the screenshot below), with Column A the dependents and Column B their respective governors.


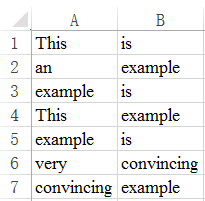


1. Data transformation. The data of an Excel form (an .xls file) should be transformed into a .net file before importing into the software **Pajek** by using a software **Creatpajek** (see the screenshot below).

**
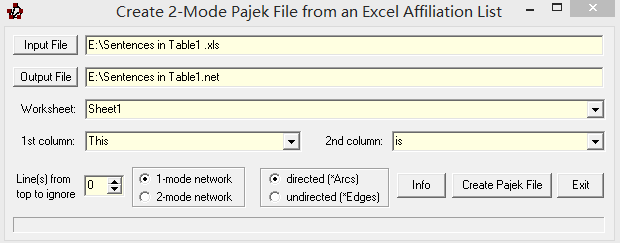
**

1. Data importation. Click “File → Network → Read” on the main window of **Pajek**, then the .net file was imported into the software **Pajek** (see the screenshot below).

**
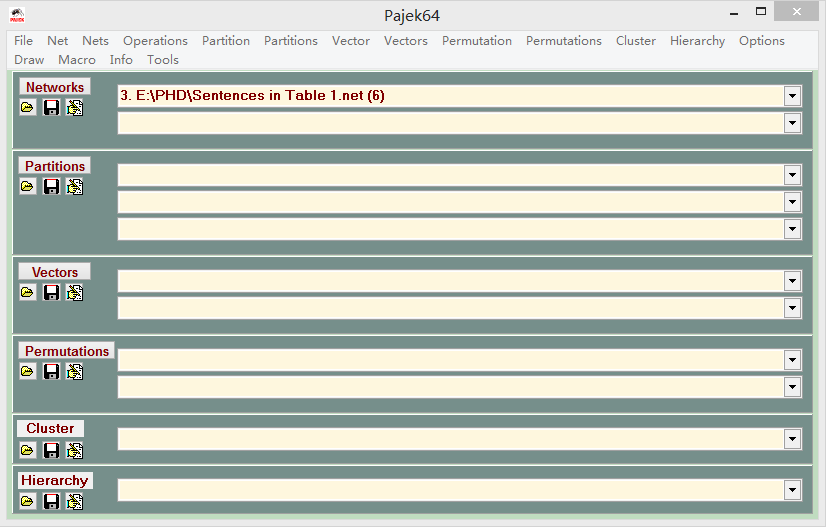
**

1. Network analysis.

Click “Net → Transform → Remove → Multiple lines/Loops” on the main window of **Pajek**, then the multiple lines and the loops of are removed from the network;

Click “Draw → Draw” on the main window of **Pajek**, then click “Layout → Energy → Kamada-Kawai → Free” on the draw window of Pajek, we could obtain a macro pictures of the network;

Click “Net → Partitions →Degree → All” on the main window of **Pajek**, then the *degree* of every vertex in the network are calculated;

Click “Info → Network → General” on the main window of **Pajek**, then the value of the network’s *average degree* could be reached;

Click “Net → Vector → Clustering Coefficient → CC1” on the main window of **Pajek** to calculate the *clustering coefficient* of the network;

Click “Net → Paths Between 2 Vertices → Distribution of Distance→ From All Vertices” on the main window of **Pajek**, the *shortest path length* of the network is obtained;

Click “Net → Vector → Centrality → Closeness → All” on the main window of **Pajek**, then the *closeness centrality* of every vertex in the network is calculated;

Click “Net → Vector → Centrality → Betweenness” on the main window of **Pajek**, the *betweenness centrality* of every vertex in the network is calculated.

**Appendix II: English explanations of Chinese function words in Table 4, 5 and 6**

| Chinese & pinyin | English | Chinese & pinyin | English |
| --- | --- | --- | --- |
| 的 (de) | of | 经过 (jing guo) | by; through |
| 在 (zai) | at; in | 被 (bei) | by |
| 和 (he) | and | 为 (wei) | by; in |
| 给 (gei) | by; to | 不 (bu) | not |
| 与 (yu) | and | 真 (zhen) | really |
| 对 (dui) | to | 都 (dou) | both |
| 用 (yong) | with; because of | 也 (ye) | too; yet |
| 就是 (jiu shi) | just | 渐渐 (jian jian) | gradually |
| 当 (dang) | when | 向 (xiang) | to; toward |
| 从 (cong) | from | 只 (zhi) | only |
| 于 (yu) | to; at; from; by | 就 (jiu) | from; simply; right away |
| 才 (cai) | just; only | 又 (you) | again |
| 以 (yi) | in order to; according to; by; with | 别 (bie) | besides; in addition |
| 因 (yin) | because of | 而 (er) | now; then |
| 只有 (zhi you) | only | 或 (huo) | or |
| 不行 (bu xing) | unfit |  |  |
